# Claudin-5, occludin, zonulin and tricellulin levels of children with attention deficit/hyperactivity disorder

**DOI:** 10.1192/j.eurpsy.2023.279

**Published:** 2023-07-19

**Authors:** H. Ferahkaya, O. F. Akca, I. Kılınç, T. Baysal

**Affiliations:** 1 Child and Adolescent Psychiatry, Dr. Ali Kemal Belviranlı Women and Children’s Hospital; 2 Child and Adolescent Psychiatry; 3 Biochemistry; 4 Pediatric Cardiology, Necmettin Erbakan University Meram School of Medicine, Konya, Türkiye

## Abstract

**Introduction:**

Accumulating studies have pointed out that gut-blood and blood-brain barrier dysfunctions due to the alterations in permeability may play a role in the pathophysiology of neurodevelopmental disorders. Tight junctions are crucial components of these barriers and some peptides including claudin-5, occludin, zonulin and tricellulin are important components of these structures.

**Objectives:**

This study aimed to investigate the relationship between these molecules and attention deficit hyperactivity disorder (ADHD) in children and adolescents.

**Methods:**

A total of 57 children with ADHD and 60 controls aged between 6 and 12 years were included in the study. The severity of ADHD symptoms was assessed through a parent-rated questionnaire and Conner’s Continuous Performance Test. Serum levels of biochemical variables were measured using enzyme-linked immunosorbent assay kits.

**Results:**

Serum claudin-5 and tricellulin levels were significantly lower in the ADHD group compared to the control group. The difference between the groups in terms of serum claudin-5 and tricellulin levels remained significant after controlling for confounding factors such as age, gender and autistic characteristics. There was no significant difference between the groups in terms of serum zonulin and occludin levels. (Table 1)

**Table 2.** Serum claudin-5, occludin, zonulin and tricellulin levels of ADHD and controls

	ADHD (n=57)	Controls (n=60)	Statistical Analysis				
				ANCOVA^c^			
	Mean ± SD	Mean ± SD	*z/t*	*p*	*F*	*p*	*η_p_^2^*
Claudin-5 *(ng/mL)	2,14 ± 0,71	2,47 ± 0,71	-3,702	P< 0,001^a^	10,196	0,002	0,083
Occludin *(ng/mL)	3,75 ± 7,17	2,99 ± 2,75	-0,136	p= 0,892^a^	0,264	0,608	0,002
Zonulin *(ng/mL)	4,82 ± 5,89	5,06 ± 5,53	-0,076	p= 0,939^a^	0,008	0,930	< 0,001
Tricellülin (ng/mL)	3,04 ± 0,56	3,34 ± 0,71	-2,552	p= 0.012^b^	6,650	0,011	0,56

**Conclusions:**

These results suggest that claudin-5 and tricellulin may be involved in the etiopathogenesis of ADHD. Alterations in these peptides may affect the brain by leading a dysregulation in intestinal or blood-brain barrier permeability that eventually affects the gut-brain axis. The causal relationship between these peptides and ADHD requires further investigation.

**Disclosure of Interest:**

None Declared

